# Infectious Complications in Patients with B-Cell Non-Hodgkin Lymphoma Treated with Bispecific Antibodies

**DOI:** 10.3390/cancers17152426

**Published:** 2025-07-22

**Authors:** Agnieszka Szymczyk, Joanna Drozd-Sokołowska, Iwona Hus

**Affiliations:** 1 Department of Hematology, National Medical Institute of the Ministry of Interior and Administration, Wołoska 137 Str., 02-507 Warsaw, Poland; iwonach.hus@gmail.com; 2 Department of Hematology, Oncology and Internal Medicine, Medical University of Warsaw, Stefana Banacha 1 A Str., 02-097 Warsaw, Poland; joanna.drozd-sokolowska@wum.edu.pl

**Keywords:** bispecific antibodies, infection, B-cell lymphoma

## Abstract

Bispecific antibodies (BsABs) have become a new standard of treatment of refractory/relapsed patients with diffuse large B-cell lymphoma and follicular lymphoma, being also intensively studied in other types of B-cell non-Hodgkin lymphoma (B-NHL). Since the therapy with BsABs results in profound B-cell depletion and T-cell exhaustion, it is associated with significantly increased risk of infections. Additional risk factors involve immune disorders caused by lymphoma itself and previous lines of therapy. In this review, we focus on the infectious complications in B-NHL patients treated BsABs, presenting their incidence in clinical trials, admittedly performed to a large extent during the COVID-19 pandemic, as well as the proposals of infection prophylaxis.

## Simple Summary

Even though infections constitute a significant complication of treatment with bispecific antibodies (BsABs), there are no structured guidelines for anti-infectious prophylaxis and treatment in the setting of therapy with lymphomas. In this review, we assess the occurrence of infectious complications in the course of BsABs treatment in patients with lymphomas. We also discuss the management strategies, like pharmacological prophylaxis of infections and prevention of hypogammaglobulinemia and neutropenia.

## 1. Introduction

Immunotherapy has emerged as one the most powerful strategies of patients with B-cell lymphoma treatment, along with decreasing role of chemotherapy. Since the Food and Drug Administration (FDA) approval of the first anti-lymphoma monoclonal antibody (MoAb) rituximab in 1997, many strategies have been developed, like different tumor-associated antigen (TAA) targeting naked MoAbs, antibody drug conjugates (ADC), immune-check point inhibitors, or T-cell engaging/redirecting therapies. The latter category includes two strategies: adaptive T-cell therapy, which are T cells genetically modified to express chimeric antigen receptor (CAR) recognizing TAA bispecific antibodies (BsABs), with one binding domain binding to TAA and the second one binding to the antigen on the effector cell, most frequently a T cell, using a CD3 complex.

**Figure 1:**
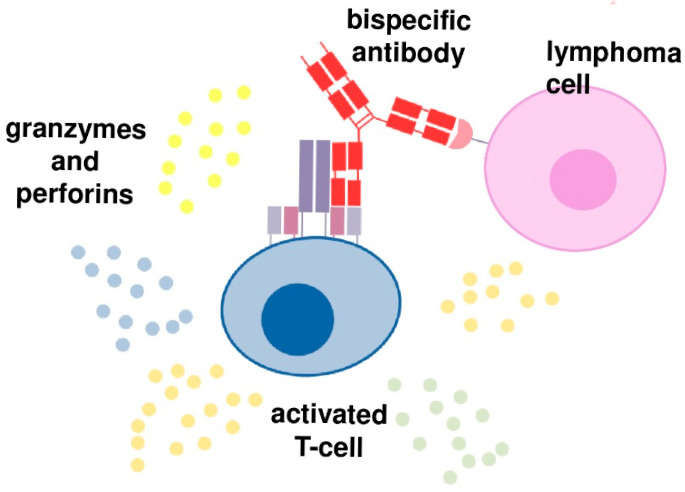
Mechanisms of action bispecific antibodies.

The concept behind two above-mentioned methods is to induce cancer cells lysis by the factors secreted by activated autologous T cells, like granzymes, perforins, and cytokines. The impulse for T cell proliferation and activation is the formation of ab immune synapse between cancer cell and T cell without the need for T cell recognition of the MHC/antigen complex on tumor cells [1] (Figure 1).

In the case of CAR T therapy, autologous T cells are obtained from the patient during leukapheresis, proliferated and activated ex vivo, and then chimeric antigen receptors are introduced using viral vectors. The immune synapse is then formed between cancer cell and CAR T cell. In case of BsABs, a patient’s own T cells are activated in vivo. BsABs have two binding domains, and the immune synapse is formed by binding to a CD3 complex expressed on T cells by one binding domain and to a B-cell specific antigen by the second one (Figure 1) [1].

Therefore, since the mode of action (MoA) of the two T-cell engaging therapies is similar, there are also similarities in their toxicity profile. The first CAR T therapies were approved in 2017, and since that time the therapy was used in more than 34,000 patients [2]. Their toxicity profile is rather well-known. The most common, clinically significant adverse events like cytokine release syndrome (CRS) and immune effector cell-associated neurotoxicity syndrome (ICANS) result from T cell activation. Long-term toxicities include increased susceptibility to infections resulting from normal B-cell depletion. The first-in class BsAB for the treatment of patients with lymphoma was approved in 2021, and though the numbers of treated patients are increasing, the experience regarding their efficacy and safety profile is still lower compared to CAR T used in clinical practice since 2017. In this review, we focus on the risk and incidence of infections in patients with B-cell lymphomas treated with BsABs, both in clinical trials as well as in clinical practice [1].

## 2. BsABs in Patients with B-Cell Malignancies

BsABs currently used in patients with B-cell malignancies target CD3 on T cell and CD20 or CD19 on B cell. BsABs targeting other antigens are under investigation in clinical trials. BsABs differ in construction, and their basic classification is based on the absence (non-IgG-like) or presence (IgG-like) of fragment crystallizable (Fc). The first-in-class BsAB was blinatumomab, approved in 2015 in European Union (EU) countries for relapsed/refractory B-cell acute lymphoblastic leukemia (ALL) and for patients with persistent minimal residual disease (MRD) after frontline therapy for B-cell ALL. Blinatumomab format is a BITE (bispecific T cell engager); it consists of two single-chain variable fragments (scFvs) targeting CD3 and CD19 antigens, without Fc portion (non-IgG-like BsAB) [3]. Therefore, blinatumomab lacks Fc-mediated effector functions, like antibody-dependent cellular cytotoxicity (ADCC), antibody-dependent cellular phagocytosis (ADCP), and complement activation. Due to small molecular size, it is characterized by high penetration to tumor tissue but at the same time by a rapid clearance from circulation. As such, it requires administration as a continuous intravenous infusion. The other “non-IgG-like” products are: bispecific killer-cell engagers (BiKEs), dual-affinity re-targeting antibodies (DARTs) and tandem diabodies (TandABs) [4]. It is worth noting that, in 2024, the first extended half-life BITE with full-length Fc was approved by FDA for patients with Small Cell Lung Cancer (SCLC) [5]. In contrast, “IgG-like” BsABs are based on the full-length IgG molecule. They have pharmacokinetic (PK) properties of MoAbs and a longer half-life [6], as well as the ability to kill cancer cell via effector functions, such as antibody-dependent cellular cytotoxicity (ADCC), antibody-dependent cellular phagocytosis (ADCP), complement-dependent cytotoxicity (CDC) [7]. However, this comes at the expense of a higher toxicity, especially CRS, as well as a risk of unwanted lysis of T cells and more difficult formation of cytolytic synapses [4].

To avoid this in newer “IgG-like” BsABs, Fc domains are modified to reduce or completely abolish binding activity to C1q and FcγR. The aim of this modification, made by using different IgG heavy chains and/or introducing specific point mutations in them (e.g., in the hinge region), is to achieve more specific T cell activation and avoid off-target toxicity by lowering activation of other immune effector cells such as macrophages and NK cells [8]. At the same time, FcRn (*neonatal Fc receptor*) binding is still preserved to maintain a longer half-life.

Another significant problem in the manufacturing process of Fc-containing BsABs that are generally encoded by two polypeptides is heavy and light chain mispairing. To solve it, different antibody platform technologies such as Knobs into Holes (KiH), CrossMab, and DuoBody have been developed [9]. There are also a new generation of BsABs with different Fc portions, such as IgG4 and IgM, and a different number of Fab regions (more antigen binding units), leading to bivalent (with 1:1), trivalent (2:1), and tetravalent (2:2) antibodies with variable avidity, stabilization of the tumor/T-cell synapse, and cytotoxic potential [10].

In the treatment of patients with B-cell lymphomas there are so far four BsABs approved by EMA and/or FDA, namely mosunetuzumab, glofitamab, epcoritamab, and odronextamab (only EMA). All of them are CD20xCD3 BsABs; they have an “Ig-like” format and an IgG1 Fc portion, except odronextamab, which has an IgG4. Their epitopes on the CD20 antigen are different, identical to rituximab for mosunetuzumab, obinutuzumab for glofitamab, and ofatumumab for epcoritamab and odronextamab. All are administered intravenously except epcoritamab, which is delivered subcutaneously. Their characteristics are presented in Table 1. All are administered using a step-up dosing (SUD) process. Mosunetuzumab and glofitamab have a fixed duration of 17 and 12 cycles, respectively, whereas the other two are currently administered until disease progression (Table 1), while administered in monotherapy [6].

There are currently (May 2025) three bispecific monoclonal antibodies approved as monotherapy for patients with diffuse large B-cell lymphoma (DLBCL)—glofitamab, epcoritamab, and odronextamab—and three approved for patients with follicular lymphoma (FL)—mosunetuzumab, epcoritamab, and odronextamab (EMA only). All BsABs in monotherapy are indicated for patients after at least two lines of previous therapy, but this will probably change in the near future, and we can expect their use in earlier lines of therapy and/or in combined protocols [11].

Mosunetuzumab is a full-length, humanized IgG1 molecule with an almost native Ab structure using KiH technology [12,13]. The first clinical trial, in which safety, tolerability, and efficacy of mosunetuzumab in patients with relapsed/refractory (R/R) B-cell non-Hodgkin lymphomas (NHL) were assessed and the recommended phase 2 dose was established, was the NCT02500407 study [14]. The study included 230 patients with R/R B-NHL. Doses up to 2.8 mg in group A and up to 60 mg in group B were assessed, respectively. The safety and tolerability of mosunetuzumab at the approved dose was assessed based on the clinical trial NCT02500407 by Budde et al. [15] in a group of 218 R/R B-NHL patients. In a pivotal phase 1/2 study, 90 patients with R/R FL and ≥2 prior lines of therapy were treated with fixed-duration mosunetuzumab. In the next clinical trials mosunetuzumab was combined with polatuzumab vedotin and R-CHOP [15].

Epcoritamab format is a DuoBody, where point mutations in the Fab portion are introduced leading to more heterodimerization and stabilization of the antibody and less T cell depletion. The pivotal trial, EPCORE NHL-1 was a multicohort, single-arm, phase 1–2 trial conducted in 128 patients with relapsed or refractory FL and in 157 patients with R/R large B-cell lymphoma (LBCL) [16].

Glofitamab is an IgG-like CD20xCD3 BsAB which possesses two CD20 binding sites in a 2:1 format [13]. It is characterized by an effector-silent Fc and three Fab arms enabling monovalent binding to CD3ɛ and bivalent binding to CD20. The second CD20 Fab is fused to the CD3ɛ-binding arm via a flexible linker to enhance functional affinity for CD20 +  lymphocytes [12]. The Fc portion is a heterodimeric IgG1-like unit that carries mutations that provide more stability and a longer half-life (10 days). In NCT03075696, the phase 2 part of a phase 1–2 clinical trial, 155 patients with R/R DLBCL who had received two prior lines of therapy were enrolled. All patients received pretreatment with obinutuzumab (1000 mg/dose intravenously 7 days before the first dose of glofitamab), followed by step-up doses of glofitamab monotherapy (12 cycles in total). Pretreatment with obinutuzumab was used to mitigate CRS. Glofitamab monotherapy was administered as a fixed-duration therapy [17]. Glofitamab combined with gemcitabine and oxaliplatin was compared in phase 3 trial with R-GemOx in 274 patients with R/R DLBCL after one or more previous therapies (Table 2) [18].

Odronextamab (REGN1979) is a human IgG4κ bispecific antibody with Fc modifications to stabilize the hinge and reduce Fc effector functions ELM-1 study [19] was the first-in-human study, single-arm, multicenter, phase 1, dose-escalation and dose-expansion study of odronextamab, where 145 heavily pretreated patients (median of 3 (IQR 2–5) previous therapies) were enrolled. The phase II study (ELM-2; NCT03888105) evaluated odronextamab in 128 patients with R/R FL and 127 with R/R DLBCL after two or more lines of systemic therapy [19]. A summary of pivotal clinical studies results is presented in Table 2.

## 3. Other Bispecific Antibodies

There are currently many studies underway on the use of new bispecific and also trispecific antibodies in lymphomas, mainly CD20xCD3, but also CD19xCD3 e.g., englumafusp alfa (clinical.trials.gov) [20]. Englumafusp alfa is an antibody-like fusion protein that simultaneously targets CD19 on B cells and 4-1BB on T cells and other immune cells. In the presence of a T-cell receptor signal and strictly dependent on CD19 crosslinking, englumafusp alfa co-stimulates T cells via 4-1BB agonism that boosts T-cell effector functions and prevents T-cell anergy [21]. Extended follow-up analysis of the dose-escalation part of phase 1 trial BP41072 has been presented recently, where patients received englumafusp alfa in combination with glofitamab. The best ORR (aNHL/iNHL) was obtained by 67.5% and 91.7% and complete response by 56.6% and 79.2% of patients, respectively [22].

## 4. Adverse Events During BsABs Treatment

T-cell activation induced by BsABs increases the risk of specific immunological complications such as CRS, ICANS, tumor flare syndrome and infections [23,24]. Cytopenias (including those of immunological origin), tumor lysis syndrome (TLS), and many other complications resulting from concurrent chemotherapy or other monoclonal antibodies have also been reported. Most patients treated with BsABs experience adverse events (AEs) ≥ grade 3, but complications of grade 5 are rare [24].

The most common manifestation of T-cell activation during T cell directing therapies (including BsABs) is CRS, observed in 45–65% of patients with B-NHL [24], but CRS grade ≥3 occurs only in 5–10% of patients [25]. The incidence of CRS is significantly higher after the initial dose and during the first treatment cycle; however, it decreases with each subsequent dose of BsABs administered [26]. It is a result of the release of proinflammatory cytokines and manifests itself as a systemic inflammatory reaction. The symptom required for diagnosis is fever ≥ 38 °C. Among the clinical symptoms are hypotension, tachycardia, hypoxia, chills, nausea, muscle aches, headaches, rash, malaise, and multiorgan dysfunction [23,25]. Interestingly CRS can occur more than once in a single patient. The management is dependent on the grade of the event. In the treatment of CRS acetaminophen, non-steroidal anti-inflammatory drugs, intravenous fluids, tocilizumab, oxygen supplementation, and vasopressors are used [23].

A less common (3–8% of patients) AE is ICANS. Its pathophysiology is not fully understood, but literature data report that it may involve inflammatory cytokine-mediated blood–brain barrier disruption [25]. ICANS manifests itself with diffuse encephalopathy symptoms, such as disorientation, consciousness disorders, apraxia, dysphasia, drowsiness, or epilepsies. In most cases, the severity is mild (grade ≤ 2) and often occurs together or after CRS [23,25,27]. Its severity is assessed using the immune effector cell-associated encephalopathy (ICE) score. In the treatment, glucocorticosteroids and antiepileptic medications are used. Tocilizumab is less effective than in CRS [23,28].

Tumor flare reactions occur most frequently after the first or the first target dose. They manifest with enlargement of the lymph nodes/infiltrative lesion accompanied by pain, erythema, and fever. In cases with lymph nodes/infiltrative lesion localized near to vital structures, such as the airways, patient monitoring is necessary. The treatment involves glucocorticosteroids [25].

Cytopenias belong to the most prevalent late complications of BsABs They occur in approximately 30–50% of all patients treated with BsABs and are more common in R/R multiple myeloma than in R/R NHL cases. Any grade neutropenia is reported in 20–40%, thrombocytopenia in 10–25%, and anemia in 15–45% of patients. Most of the cytopenias related to BsABs treatment are grade ≥ 3 [23,25]. The use of granulocyte colony-stimulating factor (G-CSF) shortens the time of neutropenia and reduces the risk of infectious complications; however, G-CSF should not be used during CRS and step-up dosing [23]. In some cases, it is necessary to transfuse platelet or red blood cell concentrate or use erythropoietin-stimulating agents [23].

## 5. Infectious Complications of BsABs

Considering the immunosuppressive effect of BsABs, it is important to keep in mind the high risk of infectious complications during therapy. It has been shown that use of BsABs targeting B cells and plasma cells leads to hypogammaglobulinemia, which is an independent factor of infectious complications. BsABs may induce T-cell exhaustion that can also increase the risk of infections [25]. Additionally, neutropenia and lymphopenia predispose to opportunistic infections and reactivation of viral infections, such as *Cytomegalovirus*, *Herpes simplex*, *Pneumocystis jirovecii* (Pj), and fungal infections. Prior immunosuppressive therapy, as well as genetic predisposition including inborn errors of immunity and comorbidities may also play a role.

Grade ≥ 3 infections occur in 15–45% of patients treated with BsABs, depending on the agent used [28,29]. It is of utmost importance to realize however, that most of the registrational trials were performed during the COVID-19 pandemic, with COVID-19 contributing significantly to the reported infections incidence. Interestingly, the real-world analysis showed lower incidence of infectious complications compared to that recorded in a clinical trial [29]. It should be emphasized that more than 70% of all reported non-relapse deaths were due to the underlying infections [30]; therefore, the analysis of the frequency and severity of infectious complications in the group of patients with lymphoproliferative neoplasms treated with BsABs is of utmost importance (Table 3).

## 6. Mosunetuzumab

In the NCT02500407 phase I/II clinical trial, cohort B (safety population), 195 AEs were recorded, including 146 treatment-related AEs (74.1%). The most frequent AEs were neutropenia (28.4%; n = 56) and infectious complications, such as urinary tract infection diagnosed in 7.6% of patients (including 2.5% grade ≥ 3) and pneumonia in 4.6% of patients (including 2.5% grade ≥ 3). Four patients died due to infections: one patient with chronic active Epstein–Barr virus (EBV) infection died from hemophagocytic lymphohistiocytosis (HLH) related to the treatment, one from sepsis, one from *Candida* sp. infection, and one from pneumonia [14].

Matasar et al. reported 182 infections (46.8% of patients, n = 102) following initial treatment with mosunetuzumab. The most common AEs were: respiratory tract infections (9.6%, n = 21), urinary tract infections (6.9%, n = 15), and pneumonias (5.5%, n = 12). Infections grade ≥ 3 were recorded in 15.1% (n = 33) of cases, and 31.7% of patients (n = 69) presented 1st or 2nd grade infections. Severe infections were observed in 17.0% of patients (n = 37), and the most common was pneumonia (3.2%, n = 7). In one case mosunetuzumab administration was discontinued due to EBV viremia. Mosunetuzumab treatment was withdrawn due to infections in 11.5% (n = 25) of patients [31]. Based on the up-to-date clinical trial NCT02500407 (median follow-up of 3.5 years—min. 2.7 years, max. 6.32 years) no new safety concerns have been reported [32]. In the next follow-up, Sehn et al. did not report any new AEs grade ≥ 3 [33].

In a dose-escalation clinical trial with expansion cohorts, JO40295, three cases of *Herpes zoster* infection (13.0%), four cases of nasopharyngitis (17.4%), and four cases of cystitis (17.4%) were reported. It was necessary to discontinue treatment due to infection (grade 3 hemorrhagic cystitis and grade 3 pyelonephritis due to adenovirus infection) in one patient. In the case of two patients (8.7%), treatment interruption was necessary; one due to *Herpes zoster* and one due to gastroenteritis in the course of CMV infection reactivation. It should be emphasized, however, that the study protocol did not refer to the use of antiviral prophylaxis, and patients in whom those AEs were reported were not on acyclovir [34].

In phase 1b/2 clinical trial NCT03671018, in the group of 120 patients with R/R LBCL, accordingly with the study protocol, mosunetuzumab was applied in step-up doses combined with polatuzumab vedotin. Treatment-related AEs grade 3 and/or 4 were reported in 38.3% of patients, and grade 5 (patients with disease progression were not included in this group) in 1.7% of patients (n = 5). Infectious complications were found in 39.2% of patients (including grade 3 or 4 in 8.3%). In 17 cases (14.2%), these were considered by the investigator as treatment-related. The most common infectious complication was pneumonia (9.2%; n = 11). In one patient, treatment was terminated due to treatment-related pneumonia, and one patient died due to pneumonia with concomitant exacerbation of heart failure. The second most common infection was COVID-19 (8.3%; n = 10). In five patients, COVID-19 pneumonia was diagnosed (including two grade 5, two grade 4 and one grade 3), and in the remaining five patients, COVID-19 without pneumonia was diagnosed (including one grade 3 and one grade 4 event). Two patients died due to COVID-19 pneumonia. Rare infectious complications included: *Candida* in 1.7% patients (n = 2), bronchopulmonary aspergillosis in 0.8% (n = 1), CMV infection in 0.8% (n = 1), EBV infection in 0.8% (n = 1), fungal infection in 0.8%, (n = 1), *Herpes simplex* infection in 0.8% (n = 1), and hepatitis B virus (HBV) reactivation in 0.8% (n = 1) of patients. Rates of infection in the dose-expansion cohort were consistent with those in the overall population (any-grade infection in 40.8% of patients, including grade 3 or 4 in 8.1% of cases), and 13.3% were considered as treatment-related [32].

In NCT03677141 phase 2 clinical trial [35], conducted in a group of 40 patients with previously untreated DLBCL, six cycles of mosunetuzumab with CHOP chemotherapy were administered. AEs were reported in all 40 patients (100%), including 20 (50%) serious AEs (SAEs). The most common any-grade AEs were neutropenia or decrease in neutrophil count, which was observed in 28 patients (70.0%), including 26 (65%) grade ≥ 3. Any-grade infections were reported in 21 (52.5%) patients. The most common infections were *Herpes zoster* (n = 5; 12.5%), pneumonia (n = 4; 10.0%), oral candidiasis (n = 3; 7.5%), and upper respiratory tract infection (n = 2; 5.0%). Grade 3, 4, or 5 infections were observed in 22.5% of patients (n = 9), including: pneumonia grade 5 due to SARS-CoV-2 infection (n = 1), pneumonia grade 3 (n = 2), aspiration pneumonia grade 3 (n = 1), *Herpes zoster* infection grade 3 (n = 1), vascular device infection grade 3 (n = 1), enterocolitis grade 3 (n = 1), influenza grade 3 (n = 1), and soft tissue infection grade 3 (n = 1). Serious infectious complications with reported concurrent neutropenia were rare—one grade 3 pneumonia occurred with concurrent grade 4 neutropenia [35].

## 7. Glofitamab

In NCT03075696, the phase 2 part of a phase 1–2 clinical trial where patients with R/R DLBCL, transformed follicular lymphoma, high-grade B-cell lymphoma, or primary mediastinal large B-cell lymphoma were included, 152 AEs were recorded. Infectious complications were observed in 38% of cases (n = 59), of which 15% (n = 23) were grade ≥ 3. The most frequently reported infection was SARS-CoV-2 infection (9%; n = 14), of which 6% (n = 9) were grade ≥ 3. The second most common infectious complication was sepsis—diagnosed in a total in 4% (n = 6) of patients [17].

Clinical trial NP30179 is a phase 1 study, investigating the clinical activity of single-agent glofitamab after single-dose obinutuzumab in patients with RR MCL. Hutchings et al. [36] reported the results of part 1 (single-patient dose-escalation) and part 2 (multiple-patient dose-escalation) of this study. Three patients were enrolled into part 1, and 171 patients were included in the second part. AEs were reported in 98.2% of patients (n = 168). Infections were observed in 51.5% of patients (n = 88), of which 17.5% were grade ≥ 3. The most common infectious complication was pneumonia, which accounted for 2.9% of cases (n = 5). Two patients discontinued treatment because of infections: one due to grade 3 CMV chorioretinitis and one due to grade 3 sepsis and grade 4 colitis [36].

The STARGLO study is a phase 3 clinical trial, which was conducted in transplant ineligible patients with R/R DLBCL, after one prior line of therapy. In this study, the efficacy and safety of glofitamab in combination with gemcitabine plus oxaliplatin (Glofit-GemOx) versus rituximab in combination with gemcitabine plus oxaliplatin (R-GemOx) was assessed. AE rates were higher in Glofit-GemOx arm (n = 180, 100% vs. n = 84, 95.5%), at the same time patients treated with immunochemotherapy in combination with glofitamab received more cycles (11 vs. 4). SAEs were reported in 17% of patients in the R-GemOx arm and 54.4% in the Glofit-GemOx arm, respectively. The most common AEs grade ≥ 3 in both groups were infections (12.5% in R-GemOx arm vs. 23.3% in Glofit-GemOx arm). Infections of any grade were reported in 29.5% vs. 57.2% of patients, including COVID-19 in 9.1% (n = 8) and 18.3% (n = 33), respectively. Death due to SARS-CoV-2 infection occurred in 3.9% (n = 7) of patients treated with Glofit-GemOx while there were no deaths reported in R-GemOx arm. Five patients (5.7%) discontinued treatment in R-GemOx arm and 22 (12.2%) in Glofit-GemOx arm due to COVID-19 [18].

## 8. Epcoritamab

In the pivotal EPCORE NHL-1 trial in patients with R/R FL [16] COVID-19 (including COVID-19 pneumonia; 51 [40%]) was the most prevalent infectious treatment-emergent adverse event (TEAE). Twenty-seven patients (21%) had COVID-19 grade 1–2, 18 (14%)—grade 3, 6 (5%) grade 5. Upper respiratory tract infections developed in 17 patients (13%), urinary tract infections in 13 patients (10%). Infectious TEAE prevalent in <10% of patients were: pneumonia (n = 10, 8%; grade ≥ 3–7, 6%), sinusitis (n = 7, 6%), *Herpes zoster* (n = 6, 5%), *Pneumocystis jirovecii* pneumonia (n = 3, 3%), pseudomonal pneumonia (n = 2, 2%), *Varicella zoster* infection (n = 3, 3%), gastrointestinal viral infection (n = 2, 2%), staphylococcal infection (n = 2, 2%). Febrile neutropenia was observed in 4 out of 128 patients (3%). Other rare infectious complications, occurring in one patient each, were: CMV colitis, CMV infection, *Haemophilus* bronchitis, *Haemophilus* pneumonia, hepatitis E, infectious pleural effusion, meningitis, pseudomonal sepsis, pulmonary tuberculosis, pyelonephritis, RSV pneumonia, septic shock, staphylococcal bacteriemia, viral gastroenteritis. Infections resulted in treatment discontinuation in 17 of 128 patients (13%): COVID-19 (including COVID-19 pneumonia) in twelve (9%) patients; hepatitis E in two (2%) patients; and pneumonia, sepsis from *Pseudomonas aeruginosa*, and sinusitis in one (1%) patient each. Fatal TEAEs were reported in thirteen patients and included infectious causes in eight cases: COVID-19 in six (5%), and pneumonia and sepsis from *Pseudomonas aeruginosa* in one patient each. Onset times to fatal COVID-19 infections ranged from 43 days to 403 days since the first dose. The exposure-adjusted incidence of COVID-19 was much higher after the emergence of omicron and subsequent variants, regardless of the epcoritamab dosing period.

Among patients with R/R FL from the Cycle 1 optimization cohort [16], COVID-19 developed in eighteen patients (21%), including in five (6%)—grade 3. Other less frequent infectious complications reported for that cohort were: *Rhinovirus* infection (5, 6%), pneumonia (4, 4%), urinary tract infection (4, 4%), bacterial skin infection (1, 1%), *Escherichia* infection (1, 1%), *Herpes zoster* (1, 1%), Pseudomonal bacteremia (1, 1%), viral pneumonia (1, 1%), febrile neutropenia (1, 1%).

In both the R/R FL and R/R LBCL cohorts of the EPCORE NHL-1 trial [37], COVID-19 was the most prevalent infectious TEAE, occurring in 30 patients (19.1%), including 13 (8.3%) grade 3 or higher. In the 2-year follow-up, COVID-19-related AEs remained the most frequent occurring in 37 patients (23.6%). Grade 3 or 4 infections were reported in 40 patients (25.5%) with LBCL; the most common (≥2.0%) were COVID-19 (8.3%), pneumonia (3.2%), sepsis (3.2%), and COVID-19 pneumonia (2.5%). The percentage of patients with grade 3 or 4 infections excluding COVID-19 was higher during the first 12 weeks of the study (10.8%) than during subsequent periods (1.9–6.7% per analysis period) [38].

Concerning combination therapies with epcoritamab, the available data on infectious complications is limited and comes mostly from EPCORE NHL-2 trial subgroups, which are presented solely in abstract form for most combinations.

Arm 4 of EPCORE NHL-2 investigated the combination of epcoritamab + R-DHAX/C in patients with R/R DLBCL eligible for autologous stem cell transplantation (ASCT). Twenty-seven patients were treated. Febrile neutropenia developed in 19% of patients, and infections in 37%, with no further details provided [39]. For unfit patients with de novo DLBCL, epcoritamab SC + R-mini-CHOP was tested in 28 patients (arm 8). One infectious grade 5 TEAE was reported, being a CMV infection reactivation [40].

In the setting of R/R DLBCL [41], 103 patients were enrolled in the study evaluating SC epcoritamab + GemOx (arm 5). Altogether, infections were among the most common TEAEs, along with hematologic AEs and CRS, occurring in 72% of the patients. Most infections were COVID-19 (29%), upper respiratory tract infection (14%), pneumonia (10%), and urinary tract infection (10%). Overall, 32% of patients experienced serious infections, most of which were COVID-19 and pneumonia. Grade 3 or 4 infections were reported in 21% of patients. Among thirteen TEAE-associated deaths, five were from COVID-19 disease. In addition to these five patients, two patients died from lung infections; both patients also had COVID-19. The remaining infectious death was due to *Escherichia coli* sepsis (n = 1). One additional death was due to enterocolitis, and one was due to multiorgan failure in the setting of acute hepatitis, with no details provided whether the conditions were of infectious origin. Most COVID-19-related deaths occurred late in treatment. Of the five patients who experienced grade 5 COVID-19, three had a CR at their last assessment. Febrile neutropenia occurred in seven patients (7%); all events were grade 3 and resolved. Importantly, the incidence of infections changed throughout the trial: in the first 12 weeks, during which the patients received epcoritamab plus GemOx, the incidence of infections was 56%; this rate decreased substantially in subsequent 12-week intervals up to week 60, to 22% to 28% for infections overall [41].

In the EPCORE-NHL2 trial in FL patients, epcoritamab was combined with rituximab + lenalidomide (R^2^) in previously untreated (1L) FL or R/R FL, or was administered as maintenance treatment [42,43]. Forty-one patients with 1L FL received epcoritamab + R^2^ (arm 6) and 20 pts in CR or partial response (PR) after standard of care treatment received epcoritamab maintenance (arm 7) [42]. In arm 6, COVID-19 was the most common TEAE (63%); two fatal infectious TEAEs occurred (COVID-19 pneumonia and septic shock). In arm 7, COVID-19 was also the most common TEAE (70%). One fatal TEAE occurred (respiratory failure; post-acute COVID-19 syndrome after 2 cycles of therapy). As mentioned above, epcoritamab-R^2^ was also administered in R/R FL (cohorts 2a/b) [43]. The 2-year follow-up update of the study was presented at ASH 2024 [44]. With most patients being enrolled and treated during the global COVID-19 pandemic, COVID-19 was reported in 57% of patients and led to epcoritamab discontinuation in 11%; COVID-19 events included five grade 5 TEAEs (COVID-19, n = 3; COVID-19 pneumonia, n = 2) [44].

## 9. Odronextamab

In the pivotal ELM-1 [45] trial infections of any grade occurred in 71 (49%) patients and included pneumonia (15, 10%), upper respiratory tract infection (14, 10%), urinary tract infection (14, 10%), and oral candidiasis (8, 6%). Grade 3 or higher infectious complications were found in thirty-three (23%) patients total, including pneumonia in twelve (8%), upper respiratory tract infection in three (2%), and urinary tract infection in two (1%) patients. Among serious TEAEs of infectious origin, pneumonia was the most common (9, 6%). Among the twelve (8%) patients who discontinued odronextamab due to TEAE, ten events were considered treatment-related and comprised CMV infection (grade 1, one patient), toxoplasmosis (grade 3, one patient), pneumonia (grade 3, two patients), and three were unrelated to treatment but also comprised infectious complications: grade 3 neck abscess in a patient with previous spinal surgery and grade 3 device-related infection. Among the four deaths considered treatment-related, two were associated with infections (lung infection after 22 days, pneumonia after 53 days), while one out of the two deaths unrelated to treatment was caused by COVID-19 after 34 days [45].

Recently the results of the CAR T expansion cohort of the ELM-1 study were published [46]. Infections occurred in 50.0% of patients, with 20.0% experiencing grade ≥ 3 infections. Infections led to treatment discontinuation in 5.0% (n = 3) of patients (COVID-19, device-related infection, and pneumonia [n = 1 each]). The rate of grade ≥ 3 infections was 21.4% in patients with high CAR-HEMATOTOX score (HT^high^) and 18.8% in those with HT^low^ during the entire study period. Compared with patients with HT^low^, patients with HT^high^ had higher rates of grade ≥ 3 infections within 30, 60, and 90 days of the first dose. Grade 5 COVID-19 pneumonia occurred in one (1.7%) patient and was considered related to treatment [46].

In the DLBCL portion of the ELM-2 study [47] infections occurred in 82/127 (64.6%) patients (grade ≥ 3, 38.6%). The most frequent type of infection was COVID-19, which was reported in 18.1% (grade ≥ 3, 12.6%) of patients. COVID-19 developed in 13 (10.2%) among 107 (84.3%) patients with grade ≥ 3 TEAEs. Febrile neutropenia was observed in three (2.4%) patients. Infections significantly contributed to treatment discontinuation. Among seventeen treatment discontinuations, six were caused by infectious causes, i.e., COVID-19, CMV reactivation, pulmonary tuberculosis, septic shock, pneumonia, and *P. jirovecii* plus neutrophil count decrease (in one patient each). Infections contributed to the death of 15 patients, including COVID-19 in 5 (4%). Other grade 5 infections included pneumonia and sepsis (n = 3 each), *P.  jirovecii* pneumonia, CMV infection, pseudomonal sepsis (n = 1 each), and CMV infection reactivation plus CMV pneumonia in one patient [47].

In the FL portion of the ELM-2 study infections occurred in 102 (79.7%) patients, including 54 (42.2%) grade 3 or higher [48]. The most prevalent type of infection was COVID-19, occurring in 47 (36.7%) patients, and 21 (16.4%) that were grade 3 or higher. Infections-associated treatment-related TEAEs that led to treatment discontinuation developed in 12 (9%) patients and apart from COVID-19 also included pneumonia, viral bronchitis, pseudomonal pneumonia, progressive multifocal leukoencephalopathy (PML) (n = 1 each). Treatment-related grade 5 TEAEs occurred in four patients (pneumonia, PML, pseudomonal pneumonia, and COVID-19 pneumonia plus systemic mycosis (n = 1 each)). Infections were observed during the treatment, with the rate of occurrence of treatment-emergent infections being stable from approximately 15 months on treatment. *Pneumocystis jirovecii* pneumonia was observed in two patients, neither of whom had received prophylaxis. In patients with severe hypogammaglobulinemia (<400 mg/dL), there was a trend toward reduced opportunistic infection in those with [1/10 (10%)] versus without [8/44 (18%)] intravenous immunoglobulin supplementation [48].

## 10. Other Bispecific Antibodies

Other bispecific antibodies are currently under investigation in clinical trials, and the information on infectious complications is frequently unavailable.

Plamotamab is a humanized CD20xCD3 BsAB. The reported data come from the first-in-human multicenter, open-label phase 1 dose-escalation study in r/r NHL patients [49]. Thirty-six patients were enrolled. In the RD (proposed recommended dosing regimen) safety population, pneumonia and sepsis occurred in 5.6% of the patients each [49].

GB261 is a novel and highly differentiated CD20xCD3 bispecific T cell engager antibody computationally designed to maintain Fc effector function, i.e., ADCC, ADCP, and complement-dependent cytotoxicity (CDC) to broaden the mechanisms of action for tumor cell killing. The compound has been evaluated in an open-label, multicenter (China and Australia), dose-escalation/expansion phase 1/2 study in patients with R/R B-NHL and chronic lymphocytic leukemia (CLL) [50]. Forty-seven R/R B-NHL patients (DLBCL: 76.6%; FL: 23.4%) were enrolled. The most common TEAE was COVID-19 infection (40.4%; grade 1 or 2: 27.6%; grade ≥ 3: 12.8%). AE-related treatment discontinuation and death were reported in two patients, which were both due to COVID-19 pneumonia [50].

Englumafusp alfa is an antibody-like fusion protein that simultaneously targets CD19 on B cells and 4-1BB on T cells. 4-1BB targeting co-stimulates T cell boosting effector cell function and prevents T-cell anergy. A phase 1b study BP41072 analyzed the combination of englumafusp alfa with glofitamab in patients with R/R B-NHL after at least one prior treatment [51]. A total of 134 patients with R/R B-NHL were enrolled, of which 83 had aggressive R/R B-NHL (60 DLBCL, 18 transformed FL, 3 transformed other indolent NHL, and 2 FL grade 3B). COVID-19 was among the most common AEs (26.9%), along with CRS, anemia, and neutropenia. A Grade 5 *Pneumocystis jirovecii* pneumonia, related to glofitamab, was qualified as a dose-limiting toxicity (n = 1) [51].

## 11. Differences in Infections Between Plasma Cell Myeloma and Lymphoma Patients Treated with Bispecific Antibodies

As summarized in the previous paragraphs, infections constitute a significant complication of treatment with CD3xCD20 BsABs; however, they are still less frequent than infections occurring in plasma cell myeloma (PCM) patients treated with BsABs especially targeting BCMA (B-cell maturation antigen). Without doubt, an increased infection risk has been observed with BsABs, as compared with conventional PCM treatment regimens [52]. In the pivotal MajecTEC-1 trial [53], treatment with teclistamab (BCMA-targeting BsAB) was associated with any-grade infectious complications in 78%, and grade 3 or higher in 52% of patients. Nearly 75% of patients suffered from hypogammaglobulinemia. In the MagnetisMM-3 trial evaluating the efficacy and safety of elranatamab, an anti-BCMA BsAB [54], infectious complications developed in 69.9% of patients, with grade 3 or higher in 39.8%. The rate of infectious complications in patients treated with linvoseltamab, another BCMA-targeting BsAB, in the LINKER-MM1 trial [55] amounted to 74.4% (33.3% Grade 3, 2.6% Grade 4); however, infection frequency and severity declined over time. The frequency of infections was significantly lower in patients treated with talquetamab, a GPRC5DxCD3 BsAB [56]. Infections occurred in 59%, 68%, and 76%, and grade 3–4 infections occurred in 20%, 18%, and 26% in the 0.4 mg/kg once a week, 0.8 mg/kg every 2 weeks, and previous TCR groups, respectively. The lower rates of high-grade infections compared with BCMA-directed therapies are explained by the limited expression of GPRC5D on B cells and normal plasma cells.

Interestingly, the incidence of grade 3 or 4 infections in RedirecTT-1 trial [57], in which talquetamab plus teclistamab were used concurrently, was higher than has been observed with either therapy alone and reached 64%.

As mentioned earlier, there are many factors which predispose patients to developing infections. They may be prevalent to different extent in PCM and B-NHL.

PCM, a disease of the immune system, is frequently associated with significant immune impairment and dysfunction of the adaptive immune response, due to malfunction of the immune regulation of plasma cells [58], which results in an increased risk of infections. Adverse events of BsABs treatment, like cytopenias or hypogammaglobulinemia, may be associated with an increased risk of infection in both PCM and B-NHL. Defects in T cell and/or B-cell immunity may predispose patients to developing opportunistic infections [52].

## 12. Discussion

Bispecific antibodies are becoming the new standard of treatment for patients with RR DLBCL and RR FL. As a monotherapy, they could be offered to the patients after ≥2 lines of systemic treatment. In April 2025, the first bispecific antibody was approved by EMA in combination with chemotherapy in DLBCL patients after ≥1 line of therapy. Currently anti-CD20 MsABs are being studied in clinical trials in earlier lines of therapy, in different combinations with chemotherapy and immunotherapy, and also in the other types of B-cell lymphoma. What is more, in 2024, the first MsAB was approved by FDA for use in small lung cancer, and other MsABs are being evaluated in pre- and clinical studies in solid tumors. Therefore, further dynamic development in this field of cancer immunotherapy is expected. In contrast to CAR T, MsABs are off-the-shelf products that can be used immediately whenever patient needs therapy, without a manufacturing process or lymphodepleting therapy. There is ongoing debate around which of these therapies is more advantageous for the patients. Pooled analysis of 16 studies comprising 1347 of patients revealed better efficacy of CAR T cells in the terms of CR and 1-year PFS and higher incidence of CRS and ICANS. There was no significant difference in infection rate [59]. The treatment with BsABs is generally well-tolerated and could be delivered on an outpatient basis in most of the cycles. However, since the therapy causes prolonged B-cell depletion, infections are a clinically significant side effect. In pivotal clinical trials with approved MsABs, the incidence of infections ranged from 50% to 78% (all grades) and 21% to 38.6% (grade ≥ 3). A pooled analysis of 2285 patients treated with BsABs identified 44% incidence of infections, including 21% infections grade ≥ 3. No difference in the infection incidence between DLBCL and FL patients and between different BsAB products was found [60]. Combining glofitamab or epcoritamab with GemOx chemotherapy did not increase the incidence of grade ≥ 3 infections [52]. However, since MsABs in monotherapy were approved basing on results of phase 1/2 clinical trials, the data from clinical practice should be carefully reported. Fatal infections occurred in 3% of patients. A significant proportion of serious and fatal infections were viral, in contrast to CAR T therapy, where most fatal infections were bacterial [52], which probably reflects the impact of the COVID-19 pandemic on the results of MsABs pivotal studies. Most of clinical trials with BsABs were carried out during the COVID-19 pandemic, so SARS-CoV-2 was responsible for not only for serious infections but also treatment-related deaths. Therefore, it is of crucial issue to properly prepare the patient and prevent infections. Before the start of lymphoma treatment or during disease remission, if possible, patients should receive prophylactic vaccines against influenza, COVID-19, pneumococcus, RSV, and zoster. Testing for Hepatitis B, C, and HIV is recommended before the commencement of treatment (CMV, EBV by PCR to be considered). Also testing for latent tuberculosis (TB) with IGRA in patients with epidemiological exposure should be performed. Pharmacological prophylaxis against *Pneumocystis jirovecii* and *Herpes zoster* should be considered and antibacterial prophylaxis is not routinely recommended. During the treatment, close monitoring for signs of infections is obligatory and immunoglobulin serum level should be regularly checked. In case of recurrent/serious infections patients could benefit with immunoglobulin supplementation (SC or IV). There are no recommendations for primary prevention of hypogammaglobulinemia and it should be considered as per institutional standards. Neutropenia could be managed with G-CSF. Except B-cell NHL and B-cell ALL, three BsABs are approved by EMA and FDA in patients with relapsed/refractory multiple myeloma (RR MM). In patients treated with anti-BCMA BsABs (teclistamab, elranatamab) infection rates are much higher than in patients with MM treated with anti-GPRC5G (talquetamab) or lymphoma patients treated with anti-CD20 BsABs: 70–80% vs. 34–47% vs. 20–45% [23]. These differences clearly show the association between target antigen on malignant cells and infection risk. Many questions regarding treatment with BsABs remain unanswered, like the optimal time of treatment, which is especially important in the aspect of immune recovery. In glofitamab pivotal trial, recovery of B-cell and IgM occurred 12–18 months after the end of treatment, and rises of IgG were noted 18–24 months after glofitamab cessation [61]. Since BsABs are still new method of immunotherapy, adverse events including infections should be reported to allow for definition of the risk of infection in clinical practice, with subsequent guidelines development on infection prophylaxis and treatment [62].

## 13. Recommendations on Infection Prophylaxis and Treatment

There are no structured guidelines for anti-infectious prophylaxis and treatment in the setting of treatment of lymphomas with BsABs. The presented recommendations summarize the general knowledge coming from both the lymphoma and multiple myeloma setting and are often based on expert opinions.

A general notion is that treatment with BsABs should be withheld in patients with active systemic infection [27]. The reason for that is the potential aggravation of immune toxicity in patients with concurrent active infection and immune cell stimulation.

Hypogammaglobulinemia can be seen in a significant proportion of patients treated with BsABs. Therefore, it is recommended to monitor immunoglobulin concentration and, if necessary, intravenous or subcutaneous immunoglobulin replacement should be offered to patients with severe or/and recurrent infections [25,27,62], as well as those with a life-threatening infection or those with documented bacterial infection with no or insufficient response to antibiotic therapy [52]. Maintaining BsABs dosing during prophylactic immunoglobulin treatment is recommended. Per analogies to plasma cell myeloma, IgG and IgM serology tests for the diagnosis of past viral infections may be used, but interpreted with caution, as patients may have received intravenous immunoglobin (IVIG), which may impact the results. Patients may also have a false negative result in response to IgG and IgM serology tests, due to a failure to mount antibody responses to pathogens. Therefore, serological status testing at baseline, although not mandatory, can be considered [52].

Both acyclovir and valacyclovir can be used for prophylaxis against the *Varicella zoster* virus. Prophylaxis should be initiated along with initiation of BsAB and maintained while the patient is receiving treatment, and thereafter for up to 6 months after treatment discontinuation. Monitoring while using these prophylactic treatments is not recommended [27,52]. B-NHL patients, if possible, should be vaccinated against Herpes zoster before initiation of BsABs; there are currently no clear data on stopping antiviral prophylaxis following vaccination.

Monitoring CMV or EBV DNA copies in B-NHL patients receiving BsABs is not routinely recommended. On the contrary, HBV screening for core antibodies and surface antigens before starting treatment in B-NHL patients is mandatory in all patients. For patients who are core antibody positive, it is recommended either to administer prophylaxis, or monitor for HBV DNA copies, with preemptive antiviral treatment for those with positive DNA tests/viremia. Patients who are surface antigen positive and/or have positive HBV DNA should receive antiviral therapy and be treated preferentially with entecavir or tenofovir under the control of specialists, as per standard treatment guidelines [27,52]. Maintaining BsAB dosing during prophylactic anti-hepatitis treatment is advisable; however, the lymphoma treatment should be discontinued if a patient experiences reactivation.

Patients treated with BsABs should receive standard vaccinations, including those against influenza and COVID-19 [27], although the response to the vaccine may be diminished. Influenza testing of nasopharyngeal or respiratory secretions is recommended only for suspected influenza. If COVID-19 is suspected, a test on nasal, nasopharyngeal, or respiratory secretions is recommended to confirm diagnosis. Following center protocols for monitoring for SARS-CoV-2 is recommended in other situations. In the case of COVID-19, patients should be treated as per local guidelines. Importantly, prolonged SARS-CoV-2 positive testing can be observed in many patients due to profound B-cell depletion [27].

General antibacterial prophylaxis is not recommended in patients receiving BsABs. In cases where it is used, the risk of development of resistant pathogens should be considered.

In patients developing bacterial infections, when the infectious agent can be identified, targeted therapy, as per standard clinical practice, should be employed. In patients with febrile neutropenia, ECIL 10 guidelines should be followed [63]. Treating microbial colonization per se is not recommended.

Current knowledge on the possible epidemiology of fungal diseases in patients with B-NHL treated with BsABs is still very limited and does not allow to suggest any recommendation apart from *P. jirovecii*. Therefore ECIL-10 recommends against antifungal prophylaxis in those patients [64]. On the contrary, as reported above, patients treated with BsABs are at risk of developing *Pneumocystis jirovecii* pneumonia. Prophylactic measures comprise trimethoprim-sulfamethoxazole, dapsone, or atovaquone. There is no data on the duration of therapy; nevertheless, the experts recommend continuing prophylaxis for up to 6 months after treatment discontinuation [27].

## Figures and Tables

**Table 1:** Characteristics of bispecific antibodies approved by EMA or/and FDA.

BsAB	Antibody Platform Technology/Half-Life (Days)	Type of Fc	Epitope of CD20	Route of Administration, Dosing Protocol	Approval (EMA/FDA)
**mosunetuzumab**	*knobs-into-holes*/6–11	IgG1	Same as rituximab	i.v.; step-up dosing, up to 17 cycles	FL (June 2022/December 2022)
**epcoritamab**	Duobody, Head-to-tail fusion/22	IgG1	Same as ofatumumab	s.c. step-up dosing, to disease progression or toxicity	DLBCL (September 2023/May 2023) FL (September 2024/June 2024)
**glofitamab**	Humanized IgG1-based 2 + 1 CrossMab/6–11	IgG1	Same as obinutuzumab	i.v.; step-up dosing, up to 12 cycles	DLBCL (July 2023/June 2023) Combined with gemcitabine and oxaliplatin (pending, positive CHMP opinion February 2025/pending)
**odronextamab**	VelociBi/14	IgG4	Same as ofatumumab	i.v. step-up dosing, to disease progression or toxicity	DLBCL (August 2024/pending)

**Table 2:** Pivotal clinical trials for BsABs approved by EMA/FDA.

	Clinical Trial Number (Phase)	Study Population/Number of Patients/Median Age (Years) F/M (%)	Number of Previous Lines of Therapy (Median)	Follow-Up (Months)	ORR/CR (%)	Median PFS/OS (Months)
**Monotherapy (≥2 prior lines of therapy)**						
**mosunetuzumab**	NCT02500407/(1/2)	RR FL/90/60/39/61	3	37.4	77.8/60	24/NR
**glofitamab**	NCT03075696/(1/2)	RR LBCL/154/66/35/65	3	12.9	52/40	4.9/50% 12-month OS: 50%
**epcoritamab**	NCT03625037 (1/2)	RR LBCL/164/64/40/60	3	25.1	63/40.1	24-month PFS/OS: 27.8/44.6 CR 65.1/76.2
	NCT03625037/(1/2)	RR FL/128/65/38/62	3	17.4	82/62.5	ND
**odronextamab**	NCT03888105 (2)	RR FL/128/61/49/51	3	20.1	80/73.4	20.7/NR
	NCT03888105 (1/2)	RR DLBCL 127/67/40/60	3	29.9	52/31.5	4.4/9.2
**Combination therapy (≥1 prior lines of therapy)**						
**Glofitamab** **+ GemOx**	NCT04408638 STARGLO (3)	RR LBCL 183/68/43/57	1	20.7	69.9/57.4	14.4/25.5

**Table 3:** Frequency of infections reported in clinical trials with bispecific antibodies.

Bispecific Antibody	Study	Infection AEs n (%)	Treatment- Related Infection AEs (n; %)	Serious Infection AEs (n; %)	Infection AEs Leading to Treatment Discontinuation (n; %)	Infection AEs Resulting in Death (n; %)	Viral Infection (n; %)	Bacterial Infection (n; %)	Fungal Infection (n; %)
**Epcoritamab monotherapy**	EPCORE NHL-1 (FL)	NR	NR	NR	n = 17; 13% (COVID-19: n = 12; 9%), hepatitis E: n = 2; 2%), pneumonia: n = 1; 1%, sepsis from Pseudomonas aeruginosa: n = 1; 1%, sinusitis: n = 1; 1%)	n =8; 6% (COVID-19: n = 6; 5%), pneumonia: n = 1; 1%, sepsis from Pseudomonas aeruginosa: n = 1; 1%)	NR	NR	NR
**Epcoritamab monotherapy**	EPCORE NHL-1 (LBCL)	Grade 3–4: 40 (25.5%)	NR	NR	NR	Treatment-related: n = 2; 1% (COVID-19 pneumonia: n = 1; 1%), bacterial pneumonia: n = 1; 1%), Unrelated to treatment: n = 3; 2% (COVID-19: n = 2; 1%), PML: n = 1; 1%)	NR	NR	NR
**Epcoritamab in combination (Epcoritamab-GemOx)**	EPCORE NHL-2 arm 5	72% (COVID-19: 29%, URTI: 14%, pneumonia: 10%, UTI: 10%); Grade 3–4: 21%	NR	32%	NR	n = 8; 8% * (COVID-19: n = 5; 5%, lung infection: n = 2; 2%, E. coli sepsis: n =1; 1%)	NR	NR	NR
**Odronextamab**	ELM-1	71 (49%) (Pneumonia: 15 (10%), URTI: 14 (10%), UTI: 14 (10%), oral candidiasis 8 (6%)); Grade ≥ 3: 33 (23%) (Pneumonia: 12 (8%), URTI: 3 (2%), UTI: 2 (1%))	NR	Pneumonia: n = 9; 6%	Treatment-related: n = 4; 3% (CMV: n = 1; 1%), toxoplasmosis: n = 1; 1%, pneumonia: n = 2; 1%); treatment-unrelated: n = 2; 1% (neck abscess in a patient with previous spinal surgery: n = 1; 1%, device-related infection: n = 1; 1%	n = 3; 2% (lung infection: n = 1; 1%, pneumonia: n = 1; 1%, COVID-19: n = 1; 1%)	NR	NR	NR
**Odronextamab**	ELM-1 (DLBCL after CAR T)	30 (50%); Grade ≥ 3: 12 (20%)	NR	NR	n = 3;5% (COVID-19: n = 1; 1.7%, pneumonia: n = 1; 1.7%, device-related infection: n = 1; 1.7%)	n = 2; 3.3% (COVID-19 pneumonia: n = 1; 1.7%, brain herniation in a patient with ongoing COVID-19 infection: n = 1; 1.7%)	n = 18; 30.0%	n = 1; 1.7%	n = 3; 5.0%
**Odronextamab**	ELM-2 (DLBCL)	82 (64.6%); grade ≥ 3: 49 (38.6%)	grade ≥ 3 TEAEs COVID-19: 13 (10.2%)	NR	n = 6; 5% (COVID-19: n = 1; 1%, CMV reactivation: n = 1; 1%, pulmonary tuberculosis: n = 1; 1%, septic shock: n = 1; 1%, pneumonia: n = 1; 1%, *P. jirovecii*: n = 1; 1%)	n = 15; 12% (COVID-19: n = 5; 4%, pneumonia: n = 3; 2%, sepsis: n = 3; 2%, *P. jirovecii* pneumonia: n = 1; 1%, CMV infection: n = 1, 1%, pseudomonal sepsis: n = 1; 1%, CMV pneumonia: n = 1; 1%)	NR	NR	NR
**Odronextamab**	ELM-2 (FL)	102 (79.7%); Grade ≥ 3: 54 (42.2%)	NR	NR	n = 12; 9% (COVID-19: n = 4; 3.1%, pneumonia: n = 2; 1.6%, COVID-19 pneumonia: n = 2; 1.6%, bronchitis viral: n = 1; 0.8%, pseudomonal pneumonia: n = 1; 0.8%, progressive multifocal leukoencephalopathy: n = 1; 0.8%, sepsis: n = 1; 0.8%)	Treatment-related: n = 4; 3% (pneumonia: n = 1; 1%, PML: n = 1; 1%, pseudomonal pneumonia: n = 1; 1%, COVID-19 pneumonia plus systemic mycosis: n = 1; 1%)	NR	NR	NR
**Mosunetuzumab**	NCT02500407 cohort B (safety population)	NR	NR	Grade ≥ 3 pneumonia: n = 5; 2.5%); urinary tract infection grade ≥ 3: n = 5; 2.5%	NR	Chronic active Epstein–Barr virus (EBV) infection and hemophagocytic lymphohistiocytosis (HLH) due to infection: n = 1, sepsis: n = 1, *Candida* sp. Infection: n = 1, pneumonia: n = 1	Chronic active Epstein–Barr virus (EBV) infection: n = 1	NR	*Candida* sp. Infection: n = 1
**Mosunetuzumab**	NCT02500407	102 (46.8%)	NR	n = 37; 17.0% (the most common of which was pneumonia: n = 9; 3.2%)	n = 25; 11.5%)	NR	Epstein–Barr virus (EBV) infection: n = 1	NR	NR
**Mosunetuzumab**	JO40295	NR	NR	0	In 1 case due to grade 3 hemorrhagic cystitis and grade 3 pyelonephritis due to adenovirus infection	NR	n = 3; 13.0% *Herpes zoster* infection; n = 1 gastroenteritis in the course of *Cytomegalovirus* (CMV) infection reactivation; n = 1 grade 3 hemorrhagic cystitis and grade 3 pyelonephritis due to adenovirus infection	NR	NR
**Mosunetuzumab**	NCT03671018 (R/R LBCL)	39.2%	n = 17; 14.2%	8.3%	Pneumonia: n = 1	Pneumonia with concomitant exacerbation of heart failure: n = 1; 0.8%); COVID-19: n = 2; 1.7%	COVID-19: n = 10; 8.3), CMV infection: n = 1; 0.8%, EBV infection: n = 1; 0.8%; *Herpes simplex* infection: n = 1; 0.8%, hepatitis B virus reactivation: n = 1; 0.8%	NR	*Candida* infection: n = 2; 1.7%, bronchopulmonary Aspergillosis: n = 1; 0.8%, fungal infection: n = 1; 0.8%
**Mosunetuzumab**	NCT03677141 (DLBCL)	21 (52.5%)	NR	n = 9; 22.5%	NR	Pneumonia due to SARS-CoV-2 infection: n =1	*Herpes zoster:* n = 5; 12.5%	NR	oral candidiasis: n = 3; 7.5%
**Glofitamab**	NCT03075696 (MCL)	59 (38%)	NR	n = 23; 15.0%	NR	NR	COVID-19: n = 14; 9%	sepsis: n = 6; 4.0%	NR
**Glofitamab**	NP30179	88 (51.5%)	NR	17.5%	n = 2; 1.2%	n = 2; 1.2%	Grade 3 CMV chorioretinitis: n = 1; 1.2%	sepsis and colitis: n = 1; 1.2%	NR
**Glofitamab**	STARGLO	57.2%	NR	23.3%	COVID-19 infection: n = 22; 12.2%	COVID-19: n = 9; 3.9%	COVID-19 infection: n = 33; 18.3%	NR	NR

* There were two additional deaths; potentially infection-related: enterocolitis (n = 1); acute hepatitis (n = 1); no further details were however provided. DLBCL—diffuse large B-cell lymphoma, FL—follicular lymphoma, LBCL—large B-cell lymphoma, MCL—mantle cell lymphoma, CMV—Cytomegalovirus, COVID-19—coronavirus disease 2019, NR—not reported, PML—progressive multifocal leukoencephalopathy, URTI—upper respiratory tract infection, UTI—urinary tract infection.
